# From Classroom to Screen: Enhancing Medical Faculty Competencies With Video-Editing Proficiency

**DOI:** 10.7759/cureus.111927

**Published:** 2026-07-01

**Authors:** Ujwala Bhanarkar, Yogesh Sontakke

**Affiliations:** 1 Department of Anatomy, All India Institute of Medical Sciences, Kalyani, Kalyani, IND; 2 Department of Anatomy, Jawaharlal Institute of Postgraduate Medical Education and Research, Puducherry, IND

**Keywords:** artificial intelligence, digital learning, educational technology, faculty development, medical education, multimedia learning, s: video editing, workshop evaluation

## Abstract

Introduction

The growing use of digital technologies in medical education has increased the demand for faculty proficiency in video editing and multimedia content creation. Video-based learning enhances student engagement and understanding. However, many educators lack formal training in video production and editing tools. This study aimed to evaluate the effectiveness of a national-level workshop designed to improve video-editing skills among medical faculty members from across India.

Methods

A hands-on national-level workshop on video editing and digital content creation was conducted for medical educators representing various institutions across India. The training covered essential video-editing techniques, including clip trimming, sound optimization, green screen removal, text addition, transitions, and AI-assisted content creation. Participants undertook pretest and posttest assessments to evaluate their knowledge gain. In addition, structured feedback questionnaires were used to assess participant satisfaction, confidence, and the perceived usefulness of the training.

Results

A total of 75 faculty members participated in the workshop, of whom 70 (93.3%) completed both the posttest and feedback evaluation. The mean test score increased from 4.8/10 in the pretest to 8.9/10 in the posttest, representing an 85.4% improvement in performance. Marked gains were observed across all assessed skill domains, including identification of video formats, use of editing tools, audio optimization, green screen removal, trimming and merging clips, text and transition effects, and color correction. Statistical analysis using a paired t-test demonstrated a significant improvement in participant performance following the workshop (p < 0.001). Feedback analysis revealed a high level of participant satisfaction, with all respondents (70/70, 100%) reporting improvement in their video-editing skills and 62/70 (88.6%) expressing confidence in applying the acquired techniques in their teaching practice. Furthermore, most participants rated the workshop highly for its relevance, clarity of instruction, and hands-on learning approach.

Conclusions

The workshop significantly enhanced participants' video-editing knowledge, skills, and confidence. Structured hands-on training programs are effective in enhancing digital competencies among medical educators and facilitating the integration of innovative multimedia resources into medical education.

## Introduction

The rapid evolution of educational technology has transformed teaching methods, particularly in medical colleges, where the complexity of the curriculum requires innovative approaches to teaching and learning. Among these, video-based learning has emerged as a highly effective instructional strategy, enabling educators to present complex anatomical concepts, clinical procedures, and theoretical discussions in a visually engaging manner [1-3]. Studies have demonstrated that the integration of multimedia into education significantly enhances student engagement and knowledge retention, making it a critical tool for modern educators [4,5].

However, creating high-quality video content requires specialized skills in video editing and production, areas where many educators face challenges due to a lack of formal training [6,7]. To bridge this gap, a national-level workshop on video editing was organized for faculty members of medical colleges across India. The workshop aimed to empower participants with essential technical skills, including video editing, sound optimization, text addition, and application of advanced effects such as green screen removal. By mastering these techniques, educators can create tailored educational videos that cater to the evolving needs of medical students [8].

The primary objective of the workshop was to enhance the participants' competence in using video-editing tools effectively to develop digital content for teaching and learning. The workshop also sought to evaluate the impact of such training on the participants' ability to incorporate these skills into their teaching practice. This evaluation was conducted through pre and posttests designed to assess knowledge acquisition, as well as structured feedback to measure the overall satisfaction and perceived usefulness of the training. This paper presents the findings from the workshop, highlighting its effectiveness in improving video-editing skills among medical educators and discussing its implications for medical education in India. The study emphasizes the importance of continuous professional development in digital tools to adapt to the demands of 21st-century education [9,10].

## Materials and methods

**Figure 1:**
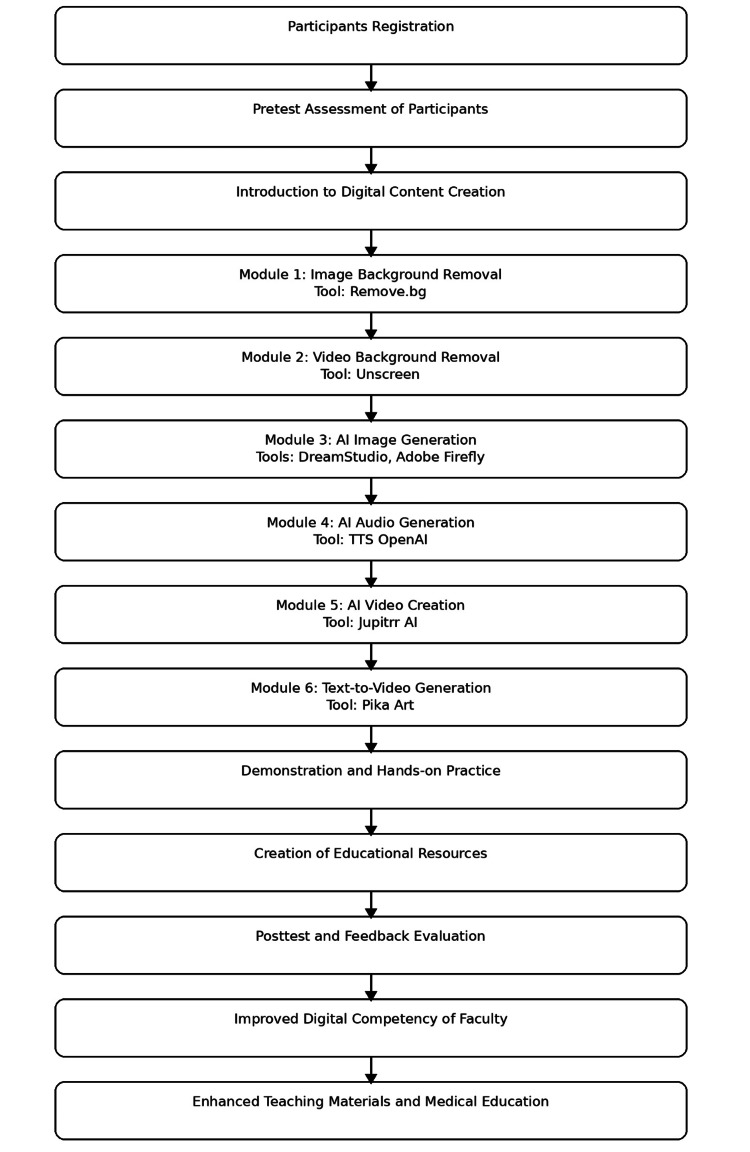
Workflow of the workshop training program on video editing and AI tools for medical educators AI: artificial intelligence

The study was conducted after obtaining approval from the Institutional Ethics Committee (IEC), AIIMS, Kalyani, West Bengal, India (Ref. No. IEC/AIIMS/Kalyani/Certificate/2025/088). A national-level hands-on workshop on video editing and digital content creation was organized at a medical college in India for faculty members from medical institutions across the country. The workshop was designed and conducted by the study authors as resource persons, who contributed to the development of the workshop curriculum, assessment tools, and evaluation framework. The workflow of the National Workshop Training Program on Video Editing and AI Tools for Medical Educators is shown in Figure 1.

The workshop was designed to provide participants with hands-on experience and practical knowledge of using video-editing software for educational purposes. It included both theoretical sessions and interactive, practical training, focusing on key video-editing techniques such as trimming clips, adjusting audio, using green screen effects, adding text and transitions, and optimizing video resolution, as shown in Table 1. Free online platforms and Adobe Premiere Pro software (Adobe Inc., San Jose, CA) were used during the training for professional video production and editing.

**Table 1:** AI tools introduced during the workshop AI: artificial intelligence

Tool	Purpose	Educational application
Remove.bg	Remove image background	Anatomy illustrations
Unscreen	Remove video background	Teaching videos
DreamStudio	Text-to-image generation	Educational graphics
Adobe Firefly	AI image generation	Presentation material
TTS OpenAI	AI voice generation	Narration
Jupitrr AI	Audio-to-video conversion	Educational videos
Pika Art	Text-to-video generation	Animated content

Participants were selected based on their interest in incorporating digital tools into their teaching practices. A total of 75 faculty members participated in the workshop: 48 (64%) females and 27 (36%) males. The sample comprised all faculty members who voluntarily registered for and attended the workshop during the study period, representing a convenience sample. These participants represented a range of medical specialties and academic backgrounds, with varying levels of experience in digital content creation. The pretest was administered at the beginning of the workshop to assess participants' baseline knowledge of video-editing tools and concepts. It consisted of multiple-choice questions and short-answer queries related to file formats, basic editing tools, sound optimization, and video effects.

The posttest, which mirrored the pretest in structure and content, was conducted at the end of the workshop to evaluate the participants' knowledge acquisition. Participants’ responses were compared to assess changes in their understanding and proficiency in video editing. In addition to the tests, a structured feedback form was distributed to participants at the end of the workshop. The feedback form included both quantitative questions on the effectiveness of the training, clarity of instructions, and overall satisfaction, and open-ended questions that allowed participants to provide qualitative feedback on their learning experience and suggest areas for improvement. 

Of the 75 participants, five were unable to complete the posttest and feedback questionnaire because they left the workshop before its conclusion due to personal or official commitments. Consequently, only the data from the 70 participants who completed both the pretest and posttest, along with the feedback questionnaire, were included in the final analysis. The pretest and posttest questionnaires used for knowledge assessment, as well as the participant feedback and evaluation form, are provided in the Appendices.

The data collected from the pretest and posttest were analyzed using descriptive statistics, including mean scores and percentage improvement. Statistical analyses were performed using IBM SPSS Statistics for Windows, Version 29.0 (IBM Corp., Armonk, NY). A paired t-test was employed to assess the statistical significance of the improvement in knowledge and skills between the pretest and posttest. The feedback data were analyzed both quantitatively and qualitatively. Quantitative feedback was summarized using descriptive statistics, while qualitative feedback was analyzed thematically to identify key trends and insights related to the effectiveness of the workshop. All data were anonymized to ensure participant confidentiality, and ethical guidelines were adhered to throughout the study.

## Results

The workshop attracted 75 faculty members (100%) during the pretest phase, with 70 participants (93.3%) completing the posttest and feedback evaluations. These participants represented diverse academic specialties, including anatomy, physiology, and clinical disciplines, with teaching experience ranging from early-career educators to seasoned faculty members. Despite their varied backgrounds, a common theme emerged from the pretest results: many participants had limited or no prior training in video editing, making the workshop highly relevant to enhancing their digital competencies. For comparative analysis, only the 70 participants who completed both the pretest and posttest assessments were included in the final analysis.

Pretest findings revealed limited familiarity with basic video-editing concepts and tools among participants. Only 18 (25.7%) participants correctly identified common video file formats such as MP4, while 14 (20.0%) could name basic editing tools such as the Razor Tool or Ultra Key. More advanced concepts, including sound optimization and green screen removal, were poorly understood, with 53 (75.7%) participants providing incorrect responses or leaving the questions unanswered. The mean pretest score was 4.8/10, indicating a substantial knowledge gap in video-editing skills.

Posttest results showed a remarkable improvement, with the average score increasing to 8.9/10, representing an 85% increase in performance. Participants demonstrated a much stronger understanding of key video-editing techniques. For example, 63 (90.0%) participants correctly identified the "Ultra Key" tool for green screen removal, a dramatic improvement from just 18.0% in the pretest. Similarly, knowledge of the "Audio Gain" tool for sound enhancement increased from 7 (10.0%) in the pretest to 62 (88.6%) in the posttest. Statistical analysis using a paired t-test confirmed that the improvement in scores was statistically significant (p < 0.001), reinforcing the workshop’s effectiveness in improving participants' knowledge and skills.

The workshop also successfully enhanced participants’ proficiency in various practical editing tasks. By the end of the workshop, over 60 (85%) participants were able to competently use tools like the "Razor Tool" for trimming clips, "Scale to Frame Size" for resizing, and "Audio Gain" for adjusting sound levels. More advanced skills, such as applying speed adjustments and color correction, saw a notable increase in competence, with 60 (85%) participants demonstrating these techniques, compared to only 10 (15%) in the pretest. This shows the comprehensive nature of the training and its positive impact on participants' ability to create high-quality educational videos.

Feedback analysis revealed overwhelmingly positive responses regarding the structure and content of the workshop. All participants (100%) agreed that the hands-on training sessions were effective and adequately met the workshop’s objectives. Many highlighted the value of real-time practice with professional software, which helped them gain confidence in using these tools. Furthermore, 67 (95.7%) participants found the instructions clear and accessible, with facilitators praised for their ability to address individual queries and guide participants through each editing step.

**Figure 2:**
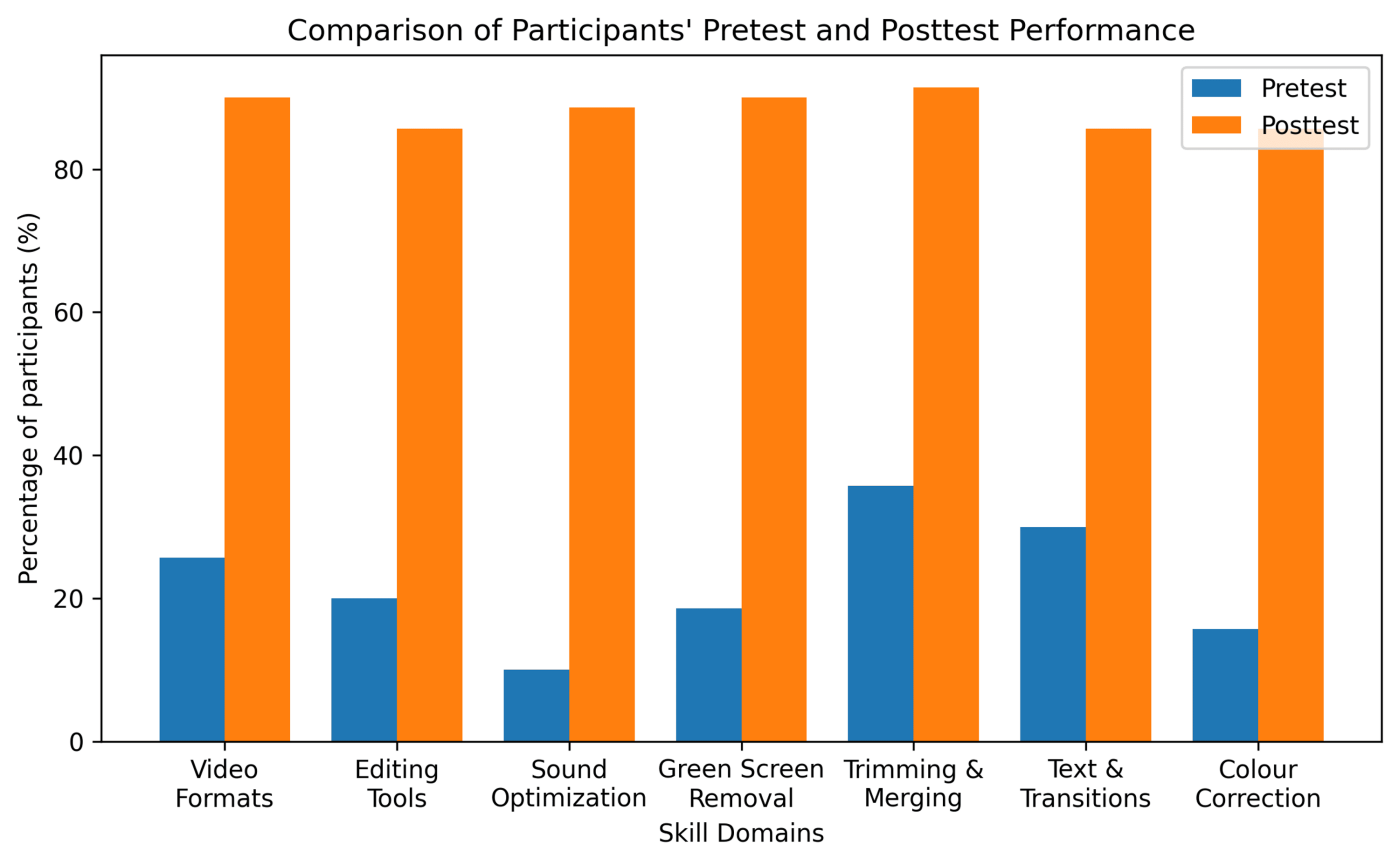
Comparison of participants’ pretest and posttest performance across key video-editing skill domains following the national workshop The Y-axis represents the percentage of participants demonstrating competency in each skill domain

In terms of skill development, 70 (100%) respondents indicated that their video-editing skills had improved significantly, with 62 (88%) reporting that they felt confident applying these newly acquired skills in their teaching practices. Most participants gained expertise in creating educational videos, optimizing audio, using green screen effects, and adding text overlays to enhance learning materials. Participants rated the overall workshop experience highly, with 63 (90.0%) awarding scores of 4 or 5 out of 5, citing the workshop as a transformative experience that equipped them with essential tools for improving their teaching (Table 2, Figure 2).

**Table 2:** Pretest and posttest performance summary Mean scores are presented as average performance scores. Improvement (%) was calculated as the difference between pretest and posttest percentages

Skill area	Pretest (%)	Posttest (%)	Improvement (%)
Identification of video file formats (e.g., MP4)	18 (25.7%)	63 (90.0%)	64.30%
Identification of basic editing tools (e.g., Razor Tool, Ultra Key)	14 (20.0%)	60 (85.7%)	65.70%
Sound optimization tools (e.g., Audio Gain)	7 (10.0%)	62 (88.6%)	78.60%
Green screen removal (Ultra Key)	13 (18.6%)	63 (90.0%)	71.40%
Trimming and merging clips	25 (35.7%)	64 (91.4%)	55.70%
Applying text and transitions	21 (30.0%)	60 (85.7%)	55.70%
Color correction and speed adjustment	11 (15.7%)	60 (85.7%)	70.00%
Mean test score	4.8/10	8.9/10	85.40%

Qualitative feedback revealed two major learning outcomes: first, participants reported a newfound ability to create engaging, high-quality educational videos for their courses; second, they gained a deeper appreciation for the potential of video editing as a tool for improving student engagement and retention (Table 3). Many suggested that the workshop could be extended to cover more advanced editing techniques, and several expressed interest in additional resources, such as tutorial videos, to continue practicing after the workshop.

**Table 3:** Participant feedback summary

Feedback category	Rating	N (%)
Hands-on training effectiveness	Excellent/good	70 (100%)
Clarity of instructions	Excellent/good	67 (95.7%)
Relevance of content	Excellent/good	70 (100%)
Confidence in applying skills	Confident	62 (88%)
Overall workshop satisfaction	4 or 5 out of 5	63 (90%)
Would recommend to others	Yes	70 (100%)

These results underscore the effectiveness of the workshop in significantly enhancing the video-editing skills of medical educators across India. The improvement in pretest and posttest scores, combined with positive participant feedback, highlights the transformative potential of such workshops in empowering educators to integrate digital tools into their teaching practices, ultimately improving the quality of medical education.

## Discussion

The results of this workshop demonstrate a significant improvement in video-editing skills among medical faculty members across India, as evidenced by the marked increase in posttest scores and overwhelmingly positive feedback. This finding aligns with previous studies that highlight the effectiveness of hands-on training in enhancing digital competencies among educators [2,11]. The significant increase in knowledge and skill acquisition is particularly noteworthy given that many participants had limited prior experience with video-editing tools before attending the workshop.

Our findings are consistent with those of Fisher et al., who found that structured, hands-on workshops in digital tool utilization led to substantial improvements in faculty members' ability to integrate multimedia in medical education [12]. Similarly, Haas et al. reported that faculty training programs focused on technology integration in teaching were successful in enhancing educators' technological literacy and comfort in using digital tools [13]. In both studies, participants exhibited considerable improvements in their practical application of digital tools, which is reflected in our results, where participants demonstrated enhanced proficiency in tasks such as trimming clips, optimizing audio, and applying green screen effects.

The improvement in participants’ scores on the posttest, particularly in advanced video-editing techniques like "Ultra Key" and "Audio Gain," mirrors findings from Devitt and Murphy and Mullen and Srinivasan, who emphasized the importance of interactive and hands-on learning for improving technical skills [14,15]. Mullen’s cognitive theory of multimedia learning suggests that combining visual and auditory elements in teaching, as video editing allows, significantly improves knowledge retention and cognitive processing. The substantial improvement in participants' abilities to apply these techniques demonstrates that the hands-on approach employed in the workshop was effective in enhancing both conceptual understanding and practical proficiency.

Furthermore, the feedback collected from participants underscores the success of the workshop in meeting its objectives. Similar to the findings of Arora et al., where medical educators reported improved confidence in using digital tools after training, the majority of our participants felt more confident in their ability to create educational videos and incorporate multimedia into their teaching [16]. The high satisfaction rate (90% rating 4 or 5 out of 5) parallels the findings of Kohan et al., who observed that faculty development programs with clear, practical outcomes lead to high satisfaction and long-term adoption of new teaching strategies [17].

Limitations of the study

The present study has several limitations that should be considered while interpreting the findings. First, although 75 faculty members participated in the workshop, only 70 completed the posttest and feedback assessments, which may limit the generalizability of the results. Second, the evaluation focused primarily on immediate learning outcomes, and long-term retention of video-editing skills was not assessed. Third, the absence of a control group prevents definitive attribution of the observed improvements solely to the workshop intervention. In addition, participant feedback was self-reported and may have been influenced by response bias. The study also focused on technical skill acquisition and did not evaluate whether these newly acquired competencies translated into improved student learning outcomes. Furthermore, the one-day duration of the workshop may not have been sufficient for mastery of advanced video-editing techniques. Finally, variations in participants’ prior exposure to digital technologies may have influenced learning gains and satisfaction levels. Future studies incorporating larger sample sizes, longitudinal follow-up, and objective educational outcome measures would provide a more comprehensive evaluation of the effectiveness of such faculty development programs.

## Conclusions

This study demonstrates that a structured, hands-on workshop on video editing and AI-assisted digital content creation is an effective faculty development intervention for medical educators. The workshop resulted in a significant improvement in participants' knowledge and skills, as evidenced by the marked increase in pretest to posttest scores, high levels of participant satisfaction, and enhanced confidence in creating educational videos using video-editing and AI-based tools. These findings highlight the value of practical, technology-oriented training in equipping medical faculty with the competencies required for contemporary digital teaching. Based on these findings, we recommend incorporating similar hands-on video-editing and digital content creation workshops into regular faculty development programs across medical institutions. Such initiatives can strengthen educators' digital competencies, promote the development of high-quality educational resources, and facilitate the integration of technology-enhanced teaching into undergraduate and postgraduate medical curricula. Future research should focus on multicenter studies with larger and more diverse participant populations to improve the generalizability of the findings. Longitudinal studies are also warranted to evaluate knowledge retention, sustained adoption of video-editing and AI-based tools in routine teaching practice, and their impact on student engagement, learning outcomes, and educational quality.

## Figures and Tables

**Figure 3:**
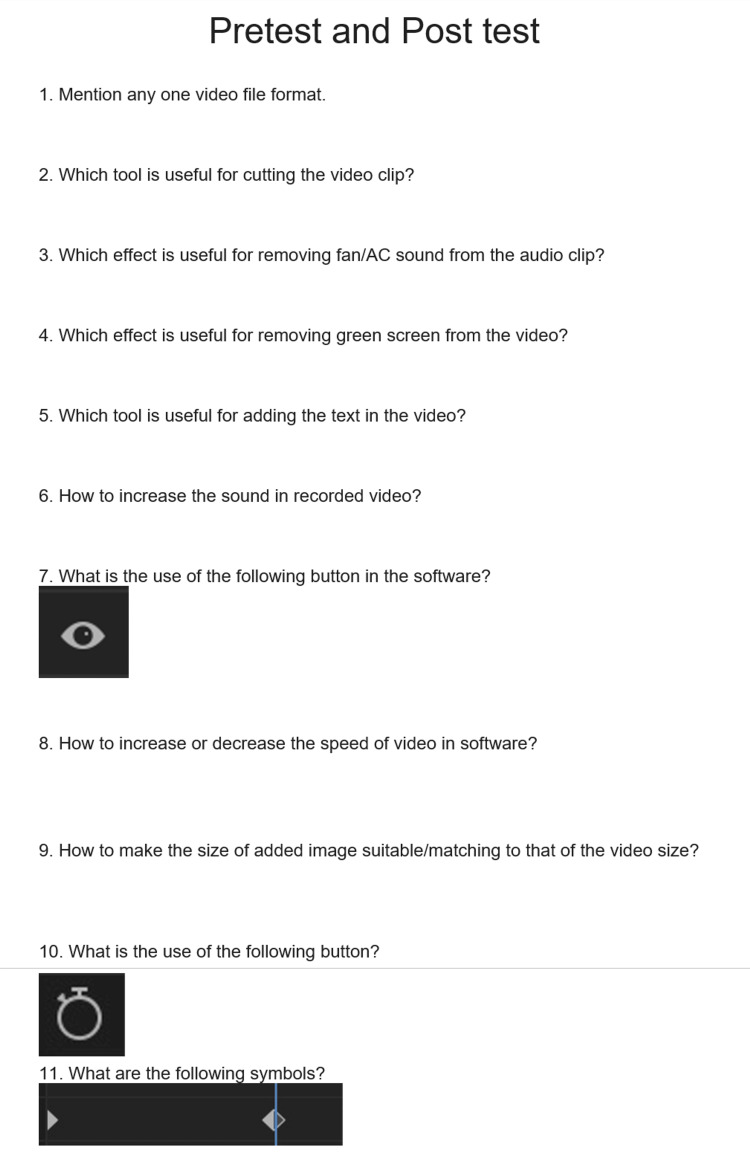
Pretest and posttest questionnaire

**Figure 4:**
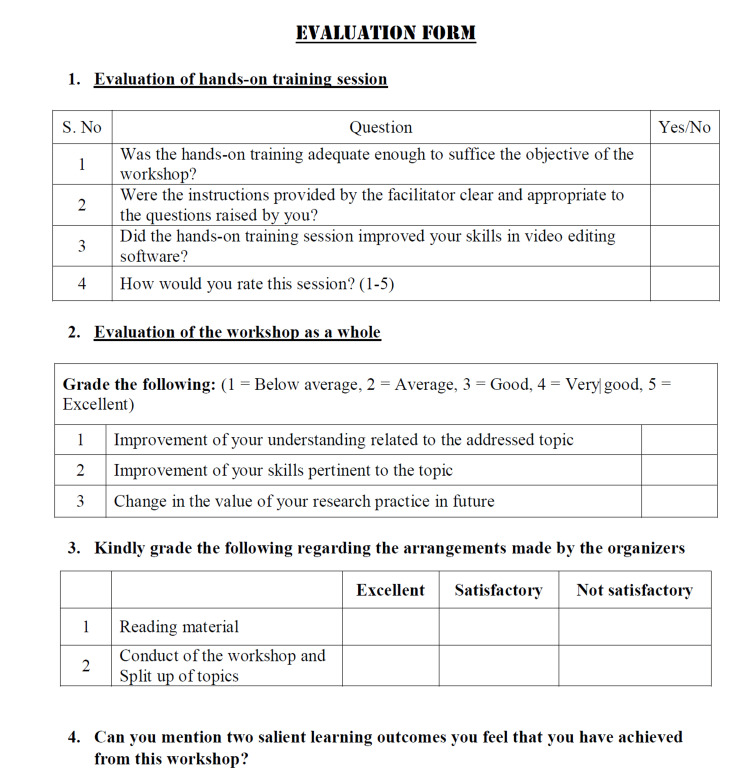
Feedback/evaluation form
